# Relative Brain Perfusion Differences in a Patient With Persistent Neurocognitive and Vestibular Symptoms Associated With Post-COVID-19 Condition: A Case Report

**DOI:** 10.7759/cureus.114380

**Published:** 2026-08-11

**Authors:** Binh Pham, Georgi Stoychev

**Affiliations:** 1 Sleep Medicine, Amen Clinics, Costa Mesa, USA; 2 Integrative Medicine, Amen Clinics, Costa Mesa, USA

**Keywords:** brain fog, brain perfusion spect, case report, dizziness, functional neuroimaging, long covid, post-covid-19 condition, single-photon emission computed tomography (spect), subjective cognitive symptoms, vestibular dysfunction

## Abstract

Post-COVID-19 condition may present with persistent subjective cognitive and vestibular symptoms despite unrevealing conventional diagnostic evaluation. We report the case of a 67-year-old woman who developed persistent subjective cognitive symptoms ("brain fog"), dizziness, vertigo, and imbalance following severe acute respiratory syndrome coronavirus 2 (SARS-CoV-2) infection. After an unrevealing conventional diagnostic evaluation, brain perfusion single-photon emission computed tomography (SPECT), obtained as part of the clinical evaluation, demonstrated a nonspecific mixed pattern of relative cortical and subcortical perfusion abnormalities. An individualized management plan was subsequently implemented, and during approximately five months of follow-up, the patient reported gradual improvement in subjective cognitive symptoms, balance, and daily functioning; however, because multiple interventions were introduced concurrently, the contribution of any individual intervention cannot be determined. This case describes a nonspecific pattern of relative brain perfusion abnormalities in a patient with post-COVID-19 condition. The relationship between these imaging findings and the patient's clinical symptoms remains uncertain, and this single observation does not establish the diagnostic or clinical utility of brain perfusion SPECT.

## Introduction

Post-COVID-19 condition is increasingly recognized as a heterogeneous clinical syndrome characterized by persistent symptoms that develop after acute severe acute respiratory syndrome coronavirus 2 (SARS-CoV-2) infection and cannot be explained by an alternative diagnosis [1]. Among the most common neurological manifestations are persistent subjective cognitive symptoms ("brain fog"), dizziness, vertigo, imbalance, and fatigue, all of which may substantially impair daily functioning and quality of life [1-3]. Despite growing recognition of these symptoms, the underlying pathophysiology remains incompletely understood.

Conventional structural neuroimaging is frequently unrevealing in patients with persistent neurological manifestations of post-COVID-19 condition despite ongoing cognitive and vestibular complaints [2,3]. Emerging functional neuroimaging studies have instead demonstrated alterations in regional cerebral perfusion and cerebral metabolism, suggesting that functional rather than structural abnormalities may contribute to persistent symptoms [4,5]. Brain perfusion single-photon emission computed tomography (SPECT) depicts the relative regional distribution of an administered radiotracer as a proxy for cerebral perfusion rather than providing a direct measurement of absolute cerebral blood flow. However, reported functional imaging findings remain heterogeneous, are not specific to post-COVID-19 condition, and have been described in a variety of neurological and systemic disorders. Consequently, the diagnostic and prognostic role of functional neuroimaging, including brain perfusion SPECT, has not been established.

Published reports describing brain perfusion SPECT in patients with post-COVID-19 condition remain limited. Available case reports and systematic reviews have identified variable patterns of cerebral perfusion abnormalities involving both cortical and subcortical regions, although interpretation is constrained by small sample sizes, methodological heterogeneity, and the absence of standardized imaging protocols [6-8]. Accordingly, the clinical significance of these findings remains uncertain.

We present the case of a patient with persistent subjective cognitive and vestibular symptoms associated with post-COVID-19 condition who underwent an extensive multidisciplinary evaluation, including brain perfusion SPECT, after conventional diagnostic studies were unrevealing. The purpose of this report is to describe the patient's clinical course, exclusionary diagnostic evaluation, brain perfusion SPECT findings, multidisciplinary management, and longitudinal clinical outcome. As a descriptive case report, this study is intended to characterize the observed clinical and imaging findings rather than establish the diagnostic, prognostic, or treatment-selection utility of brain perfusion SPECT.

## Case presentation

A 67-year-old woman presented in January 2026 with persistent subjective cognitive and vestibular symptoms associated with post-COVID-19 condition. Before SARS-CoV-2 infection, she was physically active, cognitively intact, independent in all activities of daily living, and regularly participated in recreational golf. Following SARS-CoV-2 infection in 2022, she developed persistent “brain fog” characterized by impaired concentration, short-term memory difficulties, perceived slowing of cognitive processing, difficulty multitasking, and mental fatigue. She also reported dizziness, vertigo, motion sensitivity, visual disturbances, imbalance, profound fatigue, and episodic nausea and vomiting, resulting in a marked decline in daily functioning and quality of life. She additionally described recurrent brief episodes of head pressure and an internal sensation of heat or “brain flushing,” often triggered by temperature changes or fatigue. The retrospective medical record did not clearly document the severity of the acute SARS-CoV-2 infection or the precise interval between the acute illness and onset of persistent symptoms; therefore, these details could not be further characterized.

Her medical history was notable for hypertension, prior hysterectomy, and multiple remote head injuries. In 2012, she sustained a left-sided head impact from a baseball without loss of consciousness, followed by headaches and dizziness lasting several weeks. Emergency department evaluation included unspecified imaging that was reportedly normal, although modality-specific findings were unavailable in the retrospective record. In 2014 and 2015, she sustained two additional left-sided head impacts during helmeted motorcycle accidents and reported feeling dazed for approximately 30 minutes after each event without prolonged neurological symptoms. A subsequent motorcycle fall in 2016 resulted in an X-ray reportedly demonstrating unspecified disc abnormalities. In 2024, she experienced an episode of loss of consciousness at the gym that was reportedly attributed by treating clinicians to dehydration. Before COVID-19, she remained cognitively intact and functionally independent without evidence of progressive neurological disease.

At the January 2026 evaluation, her vestibular history was notable for longstanding motion sickness with worsening dizziness, sensitivity to rapid head movements, motion sensitivity, and balance instability following COVID-19. These symptoms were characterized clinically by history; specialized vestibular testing was not performed. Sleep assessment identified difficulty with sleep initiation and maintenance, heavy snoring, and sleep bruxism, with approximately 6.5 to eight hours of sleep per night. A remote sleep study performed in the 1990s was reportedly unremarkable; no contemporary objective sleep study was available as part of the current evaluation.

Neuropsychiatric assessment further characterized her subjective difficulties with concentration, short-term memory, executive functioning, mental fatigue, and increased irritability. Brief cognitive screening with the Self-Administered Gerocognitive Examination (SAGE) yielded a score of 22/22, within the normal range. Her Patient Health Questionnaire-9 (PHQ-9) score was 4, consistent with minimal depressive symptoms, and her patient-rated Clinical Global Impression (CGI) severity score was 3, corresponding to mildly symptomatic. Formal neuropsychological testing was not performed. Neurological examination demonstrated no focal deficits.

Routine laboratory evaluation, including hematologic, metabolic, thyroid, nutritional, and inflammatory studies, did not identify a clinically significant abnormality explaining the patient's persistent symptoms (Table 1). The available retrospective record did not contain modality-specific contemporary structural neuroimaging, formal neuropsychological testing, specialized vestibular testing, or contemporary objective sleep testing sufficient to independently exclude structural, cognitive, peripheral vestibular, or sleep-related contributors.

**Table 1:** Routine laboratory evaluation Routine laboratory evaluation performed during the initial diagnostic assessment. No clinically significant hematologic, metabolic, thyroid, nutritional, or inflammatory abnormalities explaining the patient's persistent symptoms were identified. Reference ranges are those reported by the performing laboratory.

Laboratory Test	Result	Reference Range
White blood cell count	5.7 × 10³/µL	3.4–10.8
Hemoglobin	14.0 g/dL	11.1–15.9
Platelet count	277 × 10³/µL	150–450
Creatinine	0.75 mg/dL	0.57–1.00
Aspartate aminotransferase (AST)	20 IU/L	0–40
Alanine aminotransferase (ALT)	14 IU/L	0–32
Hemoglobin A1c	5.50%	4.8–5.6
Thyroid-stimulating hormone (TSH)	1.280 µIU/mL	0.450–4.500
Free thyroxine (Free T4)	1.17 ng/dL	0.82–1.77
Vitamin B12	519 pg/mL	232–1245
Folate	16.7 ng/mL	>3.0
Vitamin D (25-OH)	53.2 ng/mL	30.0–100.0
High-sensitivity C-reactive protein	<0.15 mg/L	0.00–3.00
Homocysteine	10.3 µmol/L	0.0–17.2

Given her continued functional decline, the integrative medicine team performed an expanded evaluation for potential post-infectious contributors to her symptoms. Exploratory laboratory studies included SARS-CoV-2 spike antibody testing and Epstein-Barr virus (EBV) serologies (Table 2). EBV serologies were consistent with prior exposure, with an isolated elevation of EBV early antigen IgG of uncertain clinical significance. These exploratory findings did not establish an alternative diagnosis or independently alter clinical management.

**Table 2:** Expanded laboratory evaluation Expanded laboratory studies obtained during the post-infectious evaluation. Results are presented as reported by the performing laboratory. EBV serologies were consistent with prior exposure; the isolated elevation in EBV early antigen IgG was of uncertain clinical significance. These findings did not establish an alternative diagnosis or independently alter clinical management and should not be considered diagnostic of post-COVID-19 condition or EBV reactivation. EBV, Epstein-Barr virus

Laboratory test	Result	Reference range
SARS-CoV-2 spike antibody	>25,000 U/mL	<0.8
EBV viral capsid antigen (VCA) IgG	>600.0 U/mL	0.0–17.9
EBV nuclear antigen (EBNA) IgG	491.0 U/mL	0.0–17.9
EBV early antigen IgG	25.4 U/mL	0.0–8.9

Because the available conventional evaluation did not identify a clear explanation for the persistent symptom complex, brain perfusion SPECT was obtained as an adjunctive functional neuroimaging study. The examination was performed at Amen Clinics as part of the patient's routine clinical evaluation. Image interpretation was based on visual assessment of the relative regional distribution of tracer activity displayed on the available surface-rendered brain perfusion SPECT images. Quantitative analyses, standardized normative comparator maps, orthogonal image reconstructions, and formal inter-reader analyses were not available for retrospective inclusion.

**Figure 1:**
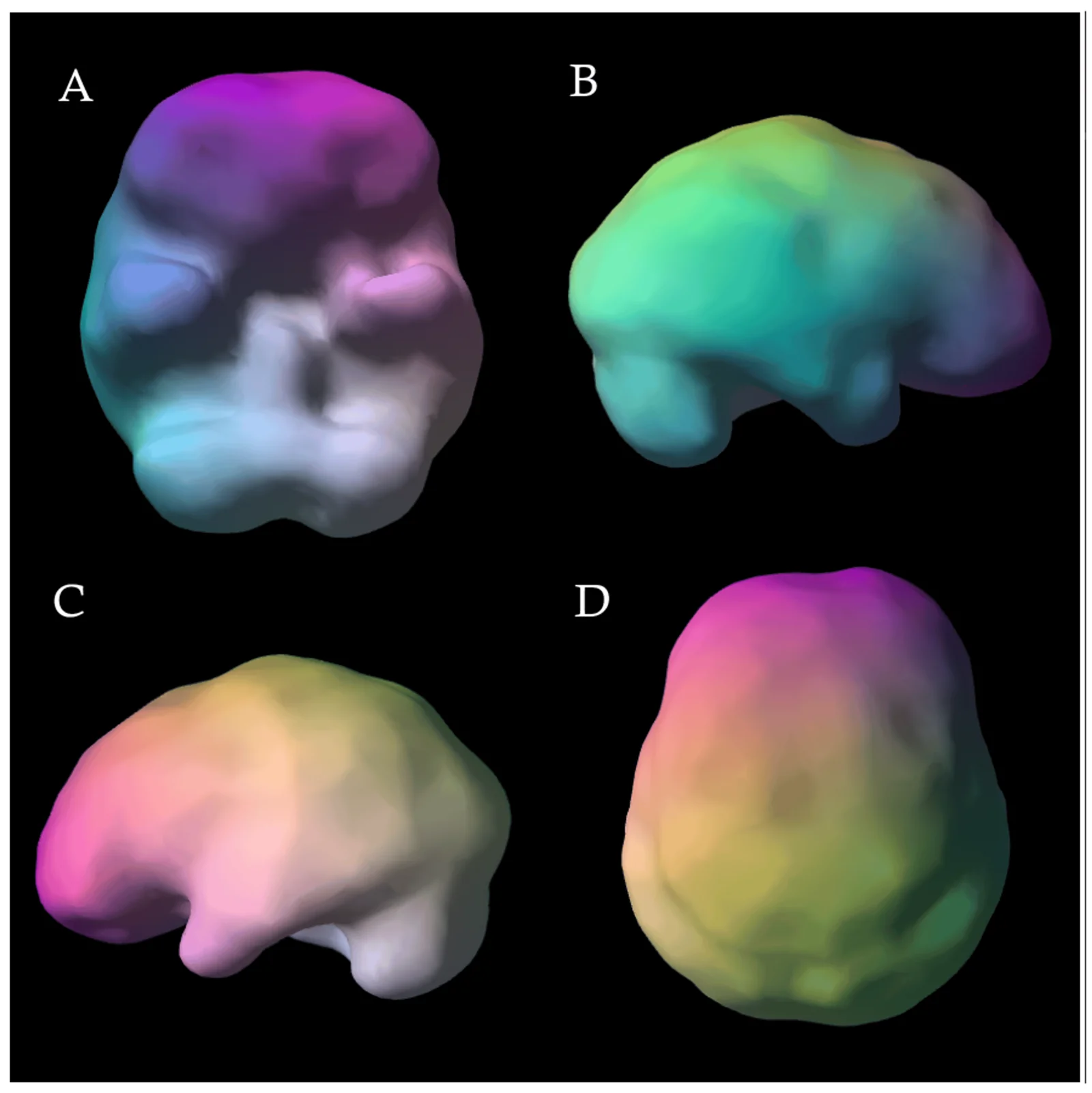
Surface-rendered brain perfusion SPECT images Surface-rendered brain perfusion SPECT images demonstrating the relative regional distribution of tracer activity. Warmer colors represent relatively greater tracer activity, whereas cooler colors represent relatively lower tracer activity within the reconstructed brain surface. (A) Inferior (underside) view. (B) Right lateral view. (C) Left lateral view. (D) Superior (top-down) view. Surface-rendered brain perfusion SPECT images demonstrate relatively increased tracer activity involving the bilateral parietal cortices, posterior cingulate gyrus, and right lateral prefrontal cortex, with relatively decreased tracer activity involving the bilateral temporal lobes, inferior orbitofrontal cortex, prefrontal poles, and internal cerebellum. The color-rendered images represent relative within-patient tracer intensity and do not provide direct absolute measurements of cerebral blood flow. A quantitative scale, documented image-normalization procedure, display threshold, normative comparator map, orthogonal sections, and corresponding quantitative outputs were not available for retrospective inclusion. Accordingly, these images are presented for descriptive and illustrative purposes and are not independently diagnostic. SPECT, single-photon emission computed tomography

**Figure 2:**
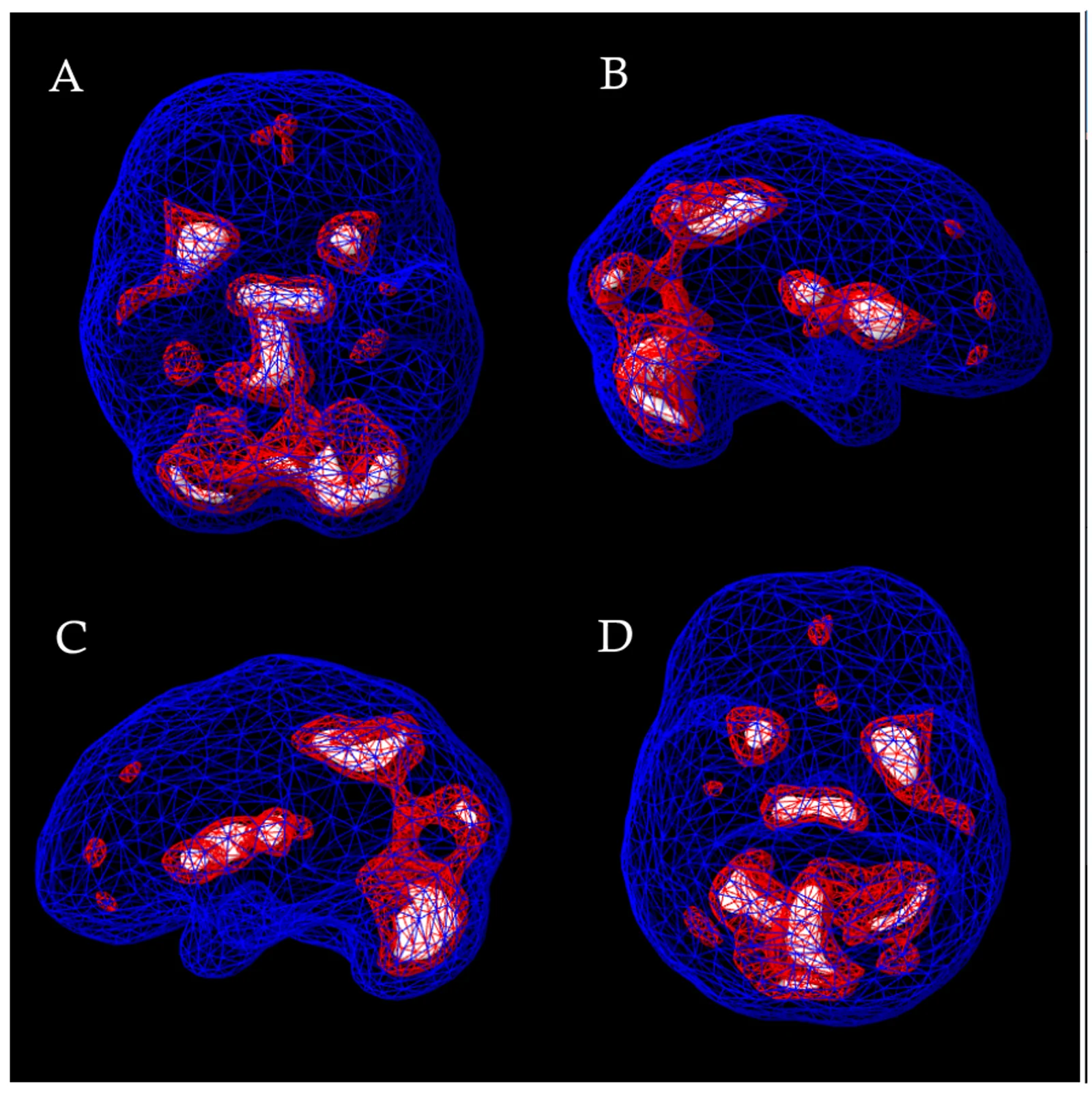
Subcortical brain perfusion SPECT images Subcortical brain perfusion SPECT images demonstrating the relative regional distribution of tracer activity. Warmer colors (white to red) represent relatively greater tracer activity, whereas cooler colors represent relatively lower tracer activity within the reconstructed brain surface. (A) Inferior (underside) view. (B) Right lateral view. (C) Left lateral view. (D) Superior (top-down) view. Subcortical brain perfusion SPECT images demonstrate relatively increased tracer activity involving the anterior cingulate gyrus, thalamus, basal ganglia, insular cortices, and the right greater than left lateral temporal lobe. The color-rendered images represent relative within-patient tracer intensity and do not provide direct absolute measurements of cerebral blood flow. A quantitative scale, documented image-normalization procedure, display threshold, normative comparator map, orthogonal sections, and corresponding quantitative outputs were not available for retrospective inclusion. Accordingly, these images are presented for descriptive and illustrative purposes and are not independently diagnostic. SPECT, single-photon emission computed tomography

Brain perfusion SPECT demonstrated relative regional perfusion abnormalities involving both cortical and subcortical structures (Figures 1, 2). Cortical surface-rendered images (Figure 1) demonstrated relatively increased tracer activity involving the bilateral parietal cortices, posterior cingulate gyrus, and right lateral prefrontal cortex, with relatively decreased tracer activity involving the bilateral temporal lobes, inferior orbitofrontal cortex, prefrontal poles, and internal cerebellum. Complementary subcortical renderings (Figure 2) demonstrated relatively increased tracer activity involving the anterior cingulate gyrus, thalamus, basal ganglia, insular cortices, and the right greater than left lateral temporal lobe. These findings represent a descriptive assessment of relative regional tracer distribution and were interpreted in conjunction with the patient's overall clinical evaluation.

Following completion of the clinical evaluation, an individualized multimodal management plan was implemented, including vestibular rehabilitation, a Mediterranean-style anti-inflammatory diet, graded aerobic and strength-based exercise, sleep optimization, nutritional supplementation, stress management strategies, and supportive care. The patient's clinical course, available diagnostic assessments, interventions, and longitudinal follow-up are summarized in Table 3. Because multiple therapeutic interventions were introduced concurrently, the contribution of any individual intervention to subsequent clinical improvement could not be determined.

**Table 3:** Clinical timeline, diagnostic evaluation, interventions, and follow-up This table summarizes the documented clinical course, available diagnostic assessments, management, and longitudinal follow-up. Exact dates were unavailable for several historical events in the retrospective clinical record; events are therefore presented according to the documented clinical sequence and approximate timing where available. Detailed acute COVID-19 severity and the precise interval between SARS-CoV-2 infection and onset of persistent symptoms were not documented. Historical imaging following the 2012 head injury was reportedly normal, although the imaging modality and detailed findings were unavailable. No formal vestibular assessment or specialized vestibular testing was performed as part of the current evaluation. Formal neuropsychological testing and contemporary objective sleep testing were also not performed. Follow-up changes in cognitive and vestibular symptoms were patient-reported. Because multiple interventions were introduced concurrently, improvement cannot be attributed to any individual intervention. CGI, Clinical Global Impression; PHQ-9, Patient Health Questionnaire-9; SAGE, Self-Administered Gerocognitive Examination; SARS-CoV-2, severe acute respiratory syndrome coronavirus 2; SPECT, single-photon emission computed tomography.

Clinical period	Clinical presentation	Evaluation and key findings	Management and outcome
2012	Left-sided head impact from a baseball without reported loss of consciousness; headaches and dizziness persisted for several weeks	Emergency department evaluation included unspecified imaging that was reportedly normal; imaging modality and detailed findings were unavailable	Subsequent treatment details were unavailable
2014–2016	Two helmeted motorcycle accidents in 2014 and 2015 involving left-sided head impacts, with approximately 30 minutes of feeling dazed after each event; additional motorcycle fall in 2016	An X-ray after the 2016 fall reportedly demonstrated unspecified disc abnormalities	No prolonged neurological symptoms were reported following the 2014–2015 injuries
2022: SARS-CoV-2 infection	SARS-CoV-2 infection preceding the persistent symptom complex	Detailed acute COVID-19 severity and the precise interval between infection and onset of persistent symptoms were not documented in the available retrospective record	Acute treatment details were unavailable
Post-COVID-19 period	Persistent brain fog, impaired concentration, short-term memory difficulty, perceived cognitive slowing, difficulty multitasking, mental fatigue, dizziness, vertigo, motion sensitivity, visual symptoms, imbalance, fatigue, nausea, vomiting, and increased irritability, with functional decline	Symptoms were patient-reported and prompted subsequent clinical evaluation	Persistent symptoms continued through the index clinical evaluation
2024	Episode of loss of consciousness at the gym	Episode was reportedly attributed by treating clinicians to dehydration; additional diagnostic details were unavailable	No further details were available in the retrospective record
January 2026: Index clinical evaluation	Persistent subjective cognitive and vestibular symptoms	Neurological examination demonstrated no focal deficits. Vestibular symptoms were documented by clinical history; no formal vestibular assessment or specialized vestibular testing was performed	Further clinical evaluation and individualized management were pursued
Sleep assessment	Difficulty with sleep initiation and maintenance, heavy snoring, and sleep bruxism; approximately 6.5–8 hours of sleep nightly	A remote sleep study performed in the 1990s was reportedly unremarkable; no contemporary objective sleep study was available as part of the current evaluation	Nightguard used nightly for sleep bruxism; sleep optimization was subsequently incorporated into management
Neuropsychiatric assessment	Subjective difficulties with concentration, short-term memory, executive functioning, mental fatigue, and increased irritability	SAGE score was 22/22, within the normal range; PHQ-9 score was 4, consistent with minimal depressive symptoms; patient-rated CGI severity score was 3, corresponding to mildly symptomatic. Formal neuropsychological testing was not performed	Clinical management and monitoring
Laboratory and SPECT evaluation	Persistent subjective cognitive and vestibular symptoms	Routine laboratory evaluation did not identify a clinically significant hematologic, metabolic, thyroid, nutritional, or inflammatory abnormality explaining the symptoms. Brain perfusion SPECT demonstrated a nonspecific mixed pattern of relative cortical and subcortical tracer distribution	SPECT findings were interpreted descriptively within the overall clinical context and were not independently diagnostic
Approximately two-month follow-up	Patient-reported improvement in brain fog, attention, memory, processing speed, balance, and overall daily functioning	Cognitive and vestibular changes were patient-reported; formal neuropsychological and specialized vestibular outcome testing was not performed	Following multimodal management, the patient resumed recreational golf, remained physically active, and reported stable mood and satisfactory sleep. Because multiple interventions were introduced concurrently, improvement could not be attributed to an individual intervention
Approximately five-month follow-up	Sustained patient-reported clinical improvement	Improvements reported at the two-month visit remained sustained at subsequent telephone follow-up	

At the two-month follow-up visit, the patient reported gradual improvement in subjective cognitive symptoms, balance, and overall daily functioning. She described that her "brain fog" had gradually lifted and reported improvements in attention, memory, and processing speed; these cognitive changes were patient-reported and were not assessed with formal neuropsychological testing. She also reported feeling significantly better overall, had resumed recreational golf after a prolonged period of nonparticipation, remained physically active, and reported stable mood and satisfactory sleep while continuing follow-up with her integrative medicine physician. At a subsequent telephone follow-up approximately three months later, these patient-reported clinical improvements remained sustained. Formal neuropsychological and specialized vestibular outcome testing was not performed.

## Discussion

Persistent subjective cognitive and vestibular symptoms are increasingly recognized manifestations of post-COVID-19 condition and may persist for months after resolution of the acute infection. Subjective cognitive symptoms, commonly described by patients as "brain fog," frequently include difficulties with attention, executive function, processing speed, and memory, whereas dizziness, vertigo, motion sensitivity, and imbalance represent common audiovestibular sequelae. Although these symptoms are well described, their underlying pathophysiology remains incompletely understood, and conventional structural neuroimaging is frequently unrevealing [1-3].

Recent neuroimaging studies suggest that alterations in regional cerebral perfusion may be associated with persistent neurological symptoms following SARS-CoV-2 infection. Using arterial spin-labeling MRI, Ajčević et al. demonstrated widespread cerebral hypoperfusion in post-COVID patients with persistent cognitive symptoms, particularly within the frontal, temporal, and parietal cortices [4]. More recently, Martins et al. reported a complex pattern of altered regional perfusion and oxygen metabolism involving the anterior cingulate cortex, insula, thalamus, and cerebellum, supporting the hypothesis that persistent neurological symptoms may reflect immune-mediated vascular and neurovascular dysfunction rather than focal structural injury [5]. Although the specific distribution of abnormalities varies among studies, the mixed cortical and subcortical tracer-distribution pattern observed in our patient is descriptively similar to previously reported functional neuroimaging findings. However, these similarities should not be interpreted as evidence of diagnostic validity because the currently available literature remains limited and heterogeneous.

Published reports describing brain perfusion SPECT in patients with post-COVID-19 condition remain limited. Tsuchida et al. reported reduced cerebral blood flow on SPECT in patients with persistent brain fog despite otherwise unrevealing conventional imaging [6]. Similarly, Ikemizu et al. described cerebellar and occipital perfusion abnormalities in a patient with post-acute COVID-19 encephalopathy whose initial presentation mimicked a primary psychiatric disorder [7]. Although the regional abnormalities reported in these studies differed from the relative tracer-distribution pattern observed in our patient, these reports collectively describe heterogeneous functional brain abnormalities involving multiple cortical and subcortical networks following SARS-CoV-2 infection. Likewise, a recent systematic review of molecular neuroimaging reported functional brain abnormalities on PET and SPECT despite relatively normal structural imaging. However, the available evidence remains limited by small sample sizes, methodological heterogeneity, and the absence of standardized imaging protocols, precluding conclusions regarding diagnostic validity or routine clinical application [8].

Several mechanisms have been proposed to explain functional neuroimaging abnormalities reported in patients with post-COVID-19 condition, including endothelial dysfunction, persistent neuroinflammation, immune-mediated vascular injury, impaired neurovascular coupling, and microvascular dysfunction following SARS-CoV-2 infection [4,5]. However, these mechanisms remain hypothetical, and no single pathophysiological pathway has been established. Accordingly, the tracer-distribution findings observed in this case should be interpreted as descriptive rather than as evidence supporting any specific underlying pathophysiological mechanism in this individual. Furthermore, regional differences in tracer distribution observed on brain perfusion SPECT are nonspecific and may be influenced by numerous clinical factors, including age, vascular disease, medications, psychiatric comorbidity, sleep disorders, and previous neurological injury. In the present case, the patient's remote mild traumatic brain injuries and hypertension represent important potential confounders. Accordingly, the imaging findings should be interpreted only within the broader clinical context and should not be considered diagnostic of post-COVID-19 condition.

This case describes relative regional differences in tracer activity on brain perfusion SPECT in a patient with persistent subjective cognitive and vestibular symptoms associated with post-COVID-19 condition. The available surface-rendered images depict relative regional tracer distribution rather than direct or quantitative measurements of cerebral blood flow. Detailed acquisition and reconstruction parameters, including the radiopharmaceutical, administered activity, patient preparation, injection-to-acquisition interval, imaging system and collimator, acquisition parameters, reconstruction and correction methods, image normalization procedure, and display threshold, were not available in the retrospective record. Accordingly, the regional observations derived from the available surface-rendered images are descriptive and are not independently reproducible from the technical information available for this report. As a single case report, these observations do not establish the diagnostic, prognostic, or treatment-selection utility of brain perfusion SPECT.

Additional limitations include the absence of formal neuropsychological testing and specialized vestibular assessment, the lack of pre-COVID-19 and longitudinal follow-up brain perfusion SPECT imaging, the absence of standardized quantitative imaging analyses, normative comparator data, orthogonal image reconstructions, and formal inter-reader assessment, as well as the lack of a comparison group. The observed tracer-distribution pattern may also have been influenced by the patient's age, hypertension, remote mild traumatic brain injuries, medications, psychiatric comorbidity, sleep-related factors, or other unrecognized confounders. Furthermore, because image interpretation was based on the available clinical surface-rendered images, the potential for interpretive bias cannot be excluded. Finally, the concurrent implementation of multiple therapeutic interventions and the possibility of spontaneous clinical recovery preclude attribution of improvement to any individual intervention. Prospective studies incorporating standardized clinical phenotyping, objective cognitive and vestibular assessments, quantitative neuroimaging methodologies, and longitudinal follow-up are needed to better define the potential clinical value of functional neuroimaging in patients with persistent neurological manifestations of post-COVID-19 condition.

## Conclusions

Persistent subjective cognitive and vestibular symptoms associated with post-COVID-19 condition remain diagnostically challenging, particularly when conventional structural imaging is unrevealing. This case documents a nonspecific mixed pattern of relative cortical and subcortical perfusion abnormalities identified on brain perfusion SPECT in a patient with persistent subjective cognitive and vestibular symptoms. However, the relationship between these imaging findings and the patient's clinical symptoms remains uncertain, and this single case does not establish the diagnostic, prognostic, or management utility of brain perfusion SPECT. Prospective studies incorporating standardized clinical phenotyping, quantitative neuroimaging methodologies, and longitudinal follow-up are needed before the potential diagnostic or clinical role of brain perfusion SPECT in post-COVID-19 condition can be defined.
